# A good resource for parents, but will clinicians use it?: Evaluation of a resource for paediatric end-of-life decision making

**DOI:** 10.1186/s12904-016-0177-5

**Published:** 2017-01-25

**Authors:** Clare Delany, Vicki Xafis, Lynn Gillam, Jo-anne Hughson, Jenny Hynson, Dominic Wilkinson

**Affiliations:** 1 Children’s Bioethics Centre, Royal Children’s Hospital Melbourne, 50 Flemington Rd, Parkville, VIC 3052 Australia; 2 University of Melbourne, Parkville, VIC 3010 Australia; 3 Sydney Children’s Hospitals Network, Children’s Hospital at Westmead, Locked Bag 4001, Westmead, NSW 2145 Australia; 4 Centre for Values Ethics and the Law in Medicine, University of Sydney, Medical Foundation Building K25, Camperdown, NSW 2006 Australia; 5 Oxford Uehiro Centre for Practical Ethics, Faculty of Philosophy, University of Oxford, Suite 8, Littlegate House, St Ebbes Street, Oxford, OX1 1PT UK

**Keywords:** Health communication, Inter-professional communication, End of life care, Consumer health information, Decision making, Paediatrics

## Abstract

**Background:**

Communication with parents about end-of-life care and decisions is a difficult and sensitive process. The objective of the present study was to ascertain clinicians’ views on the acceptability and usefulness of a handbook and web-based resource (*Caring Decisions*) that was designed as an aid for parents facing end-of-life decisions for their child.

**Methods:**

Qualitative interviews were conducted with a range of health professionals who provide care to children facing life-limiting conditions.

**Results:**

Data analysis confirmed the acceptability and usefulness of the resource. Two major themes were revealed: 1. *Family empowerment*, with sub-themes *Giving words and clarity*, *Conversation starter*, ‘*I’m not alone in this*’, and *A resource to take away*, highlighted how the resource filled a gap by supporting and enabling families in a multitude of ways; 2. *Not just for families*, with sub-themes *A guide for staff*, *When to give the resource?*, *How to give the resource* and *Who should give the resource?*, explored the significant finding that participants viewed the resource as a valuable tool for themselves, but its presence also brought into relief potential gaps in communication processes around end-of-life care.

**Conclusion:**

The interview data indicated the positive reception and clear value and need for this type of resource. However, it is likely that successful resource uptake will be contingent on discussion and planning around dissemination and use within the health care team.

## Background

### End of life decisions for newborn infants and children

Despite advances in medicine and medical technologies, a small number of children each year are diagnosed with life-limiting illnesses. Families face the prospect of their child dying in the short or medium term, and often must make extremely difficult decisions about treatments for their child. There is widespread professional and legal acceptance that it is ethical to withdraw or withhold life-sustaining medical treatments if these are not in the child’s or infant’s best interest [1–5]. However, such decisions can lead to extremely challenging discussions in the intensive care unit, in the paediatric inpatient ward, or in the outpatient or community setting. Models of clinical communication emphasise the importance of shared decision-making [6] and family-centred care where information is shared openly and collaboratively enabling decisions to be made in the best interests of the child within their family [7]. Studies have also confirmed that parents prefer the shared-decision making model [8].

### Impact on families

The experience of having a seriously ill child is highly traumatic for parents and families [9, 10]. Family members of children who have died in ICU are at risk of long-term grief and post-traumatic stress, and those who perceive that they were given inadequate information regarding end-of-life decisions are especially at risk [11–13]. They are reliant on medical staff to provide facts and advice, but also actively seek support from other sources including the internet and printed materials [14, 15]. In past surveys, one third of parents felt that they had not received adequate information about the pros and cons of continuing or withdrawing treatment [15, 16], and nearly one quarter indicated that they would have made decisions differently in retrospect [15].

### Ethical complexity in end-of-life decision-making

Professional guidelines have endorsed a partnership in care between parents and health care professionals in critical decisions [4, 5, 17] and published research has identified ethical deliberation and shared decision-making as being fundamental to such partnerships in the form of family-centred care [18]. However, the complexity of end-of-life decisions for children poses extra challenges to communication and sharing decisions. Evidence from interviews with parents suggests that they want to be part of end-of-life decisions and that they do not suffer adverse psychological consequences from their involvement [17]. Studies also suggest, however, that parents vary in the degree of involvement and responsibility they seek when making end-of-life decisions [8, 17]. Verkerk et al. (2015) have recently described an ‘ethics of families’, to highlight the moral importance of adopting a relational ethical approach to acknowledge the relationships between family members including the values parents hold about life and death for their child [19].

Adding to the complexity of sharing information when making end-of-life decisions is the fact that at times, there may be some urgency or perceived urgency about treatment decisions [20, 21] and at other times, the prognosis is uncertain and decision making is made over a longer period of watching and waiting [20]. For example, decisions to withdraw or withhold treatment are often based on the results of neuroimaging [22], yet the evidence linking neuroimaging evidence with long-term prognosis is sometimes of poor quality [23]. Parents struggle to make decisions in the face of such uncertain correlations and information.

### Support for end of life decision-making

The use of written materials enables families to access language and concepts [24, 25] and can provide one potential way of building decision-making literacy in families [26]. Provision of written informational materials encouragesing parents to speak with each other and participate in otherwise clinically-dominated decision-making environments. Written resources in health care settings are valued by parents [27] because they can clarify and help them to remember medical information [28] including discussions about medical diagnoses and prognoses [29, 30].

There is limited evidence, however, about what kind of written materials would best support parents who face decisions about life-sustaining treatment for their critically ill newborn infant or child [26]. There is a small number of existing resources for parents involved in end-of-life decision-making [31–33], but the availability of resources to help parents understand the complex and difficult ethical questions concerning their child or assist them to participate in conversations in written or web-based form are limited [34].

In 2015, Xafis and colleagues published on the development and pilot evaluation of a written resource, *Caring Decisions* handbook and web-based information [34, 35], designed to assist parents to better understand these complex decisions. The resource sought to address identified gaps and issues in existing resources on end-of-life care, and generated very positive feedback from the multidisciplinary consultation panel involved in its evaluation. Clinicians and parents in the pilot study described the resource as both helpful and comforting. It was also seen to be a useful training tool for both trainee clinicians and experienced clinicians.

Whilst this endorsement is encouraging, use of such materials relies on clinicians telling families about the resources, deciding when is the right time to raise the issue [35] and working within the treating team to identify who is best placed to initiate conversations and make decisions. Barriers to conducting advanced care discussions include clinicians’ perceptions of parents’ readiness to receive information and their prognostic understanding [36] and the need for high levels of interdisciplinary collaboration [37]. Clinicians (intensive care specialists, nurses, social workers and counsellors) are often the gatekeepers of this type of information and as part of their clinical judgement they decide when and how to have conversations and make information available to parents and include them in decision-making [38–40]. This means that resources and guidelines which are aimed at enhancing parents’ capacity to be included within the communicative culture of multidisciplinary care must be endorsed at a deeper level within clinical teams, rather than solely because they have theoretical/practical value for parents [41]. The clinicians must feel comfortable with the resource, and feel that it will be a help rather than a hindrance in their communication with the family.

## Methods

### Study aims

The goal of the current research was to gain more in-depth feedback about the acceptability and practical value of the *Caring Decisions* handbook from the perspective of a range of health professionals who are likely to be involved in caring for children whose parents will need to make end-of-life decisions and use this resource (*Caring Decisions* Handbook).

The three specific research objectives were to ascertain:

1. How clinicians involved in the care of critically ill children who face end-of-life decisions feel about the parent handbook and web-based resource;
2. Whether clinicians think the handbook would be helpful in their conversations with parents and;
3. Whether they would be willing to provide a copy of the resource to parents.

### Study design

A qualitative methodology drawing from the social theory of symbolic interactionism [42] was used to explore participants’ perspectives on whether and how they would use the handbook as part of their communication with families. Symbolic interactionism provides a theoretical framework to explore interpretive understanding of context and interactions [43]. An interview guide was developed which comprised questions concerning participants’ roles in caring for critically ill children, what they thought of the resource, how it would fit within their usual interactional approach to discussing end-of-life issues with parents, and whether they thought parents would find the resource useful. The interview guide is reproduced in Appendix 1.

### Study setting, sampling and participants

We purposively recruited health professionals who were closely involved in clinical decision-making and/or provision of care to parents of children who face life-limiting illnesses at the Royal Children’s Hospital in Melbourne, Australia. Potential participants were invited by email to their department. Participants replied by e-mail to CD. Snowball sampling [44] was also used and this involved participants informing CD of other colleagues who were involved in working with families with critically ill children and these participants were contacted directly by CD by e-mail. Ethics approval was obtained for the current research from the Royal Children’s Hospital Ethics Committee. Participants provided informed consent prior to participation.

### Data collection and analysis

Semi-structured interviews were conducted by CD from November 2014 to June 2015. During this period, CD worked as a clinical ethicist at the hospital and knew some of the participants through prior clinical ethics consultations. In accordance with the principles of researcher reflexivity [45] , CD emphasised the purpose of the interview to be about the participants’ interpretation of the caring decision resources rather than a broad discussion about clinical ethics. Participants were provided with a copy of the *Caring Decisions* handbook two to seven days prior to their interview to give them an opportunity to read through it and make notes if desired. All interviews were one-on-one, and lasted between 30–40 min. One interview comprised three participants to accommodate their work commitments. All interviews were audio-recorded, except one interview where the recording equipment malfunctioned. In this case, the interviewer took detailed notes. Reflecting the iterative nature of qualitative interviewing [46], more emphasis was given to participants’ individual work circumstances to account for their particular professional role in working with families over the interview period. The final number of interviews was determined when ideas being raised by participants were well represented by the developed themes and no new codes were being developed. [47].

Interviews were fully transcribed by CD. JH and CD analysed the transcripts using a thematic approach [48]. This process was inductive and entailed the two authors working with the data first independently, reading and re-reading transcripts to identify ideas being raised by participants. Inductive content analysis relies on developing categories and thematic description from the data, rather than reading the data with pre-determined frameworks of knowledge [49]. These broad patterns identified in the first readings were then aggregated as codes. CD and JH met monthly over a period of 4 months to dicuss how the codes could be developed into overall themes. Following each discussion, the notes and ideas were sent via e-mail to all authors for further analysis and contribution. Eighteen health professionals agreed to participate in the study: 5 physicians (2 neonatologists, 1 cardiologist, 1 paediatrician, 1 paediatric intensivist) with an average of 21 years’ experience, 9 nurses (4 paediatric intensive care unit (PICU) nurses, 1 organ and tissue donation nurse coordinator, 3 clinical nurse consultants) with an average of 18 years’ experience, 2 educational play therapists (with 5 years’ experience each), 1 chaplain (8 years’ experience) and 1 social worker (15 years’ experience).

## Results

The qualitative interview data provided direct answers to the specific research objectives: 1) All interviewees felt the handbook and web-based resources were highly valuable as an adjunct to their communication. 2) Almost all participants thought the handbook would provide information which parents may not have considered, and that the information was pitched at an appropriate reading level for parents. Many commented that a particularly valuable aspect of the handbook was that parents could take it away with them and read it a time which was suitable for them. 3) All of the interviewees expressed a willingness to provide a copy to parents.

Analysis of the interview data revealed additional and more detailed information that contextualises and builds on our understanding of how written material in the form of a handbook and web resources might be used in a complex and busy tertiary hospital setting. Two main themes emerged, each with several sub-themes: ‘Family empowerment’, and ‘A resource for staff’.

### Theme 1: Family empowerment

All of the interviewees discussed a general lack of resources available for parents needing to consider withdrawal or withholding of life support treatments for their child, and many of the participants considered that the provision of a resource such as the *Caring Decisions* handbook and web resource would be an effective way to empower parents. A sub-theme *Giving words and clarity* (see Table 1 for detailed quotes) explains how the resource could do this by ‘giving a language’ and providing a context from which to deal with a subject matter that may be entirely new and overwhelming for a parent. Many interviewees felt this language aspect to be one of the strongest elements of the resource because it enabled parents to be included in the decision-making process. The second sub-theme of *Conversation starter* focused on the practical function of these resources as enablers of difficult discussions by creating a shared language about end-of-life care decisions between parents and clinicians and making it easier for clinicians to start conversations. The third sub-theme, ‘*I’m not alone in this*’, highlighted the supportive function of the resource, to help families take comfort in the knowledge that others have been through and understand their experiences. The fourth sub-theme, *A resource to take away*, described a further potential benefit of the booklet to be its physical concreteness and portability. Participants discussed how parents may be in a state of deep shock and trauma during meetings where the healthcare team is talking about end-of-life decisions for their child. Having something that they can refer to later and keep coming back to as needed was considered to be very valuable for parents.

**Table 1:** Theme 1: Family empowerment

**Sub-theme 1: Giving words and clarity** I guess I feel like it does break it down a bit more and it gets them thinking about the types of things that may be asked of them or the types of questions that they might be asked to think about because families will often say, “You know, I’m sure I have a million questions but I don’t know what they are” so this kind of helps give words to what some things that might be on their mind and I guess it empowers them to think that they can be curious in this process and they don’t just have to take the information and they can actually ask more questions (Group interview) You know, they’re trying to understand these things that are far-fetched and unusual to them and unheard of and so giving them something they can sort of hold on to sometimes might kind of help them kind of ground themselves (Organ and tissue donation nurse coordinator) I guess it’s helping people make their own clarity about what’s actually happening here. (Chaplain) Definitely, bridges the gap a little bit and maybe gives people the chance to actually think if there’s a publication about it, it’s official? (Educational play therapist) I think it would help clarify some of the things we’re trying to talk about. (PICU nurse) … I asked her, “I’m sure you have questions, you probably don’t know what they are” and she said, “Yeah, that’s right. My head’s swimming” and I think that just seeing that page would be really helpful to streamline some of their ideas. (Group interview)	**Sub-theme 2: Conversation Starter** I like on the online version the language of like how can you start a conversation, how can you answer question and the questions that were tab options on there I thought were really useful and I can imagine myself asking those questions (as a parent) (Educational play therapist) it could be a good conversation starter a good introduction to some of the things that they were feeling, (Group interview) I think it could help open up conversations for people who may be feel a bit less confident. (Group interview) It’s a conversation starter. And then people can find their own language. (Paediatric intensivist)	**Sub-theme 4: A resource to take away** In these meetings [parents] get a lot of information, a lot of very distressing, very new and unfamiliar information and you kind of tell sometimes, they’re not really taking it all in and you never know what they’ve heard and what they haven’t heard. So I think they can sometimes really shut off about, like they just don’t want to hear the technical things. They just know the child’s going to die, they don’t need to hear how it’s going to happen sometimes but I think having something they can read, it might help them to understand the process a bit better. (Organ and tissue donation nurse coordinator) The written format enables someone to go back and review. People will always find it difficult to take everything in one go, especially if they’re very emotional. These conversations are sometimes difficult and they will forget content and this is opportunity to go back and read it again and read it again and, that’s why written information’s helpful. (Cardiologist) It doesn’t particularly say anything new or anything that we wouldn’t tell parents anyway but I think the beauty of it is that it gives them something to take away and, you know, with any of the sessions that we have with our families, the information is so complex and there’s so much of it that I don’t really expect them to take more than about 10 to 20% of it away in their minds, which is why we have these meetings repeatedly but I see enormous value in them being able to take something away and read quietly later because I think a lot of what’s written in the book would trigger memories about what was said in meetings that would allow them to feel more comfortable with the decision at a time when they’re drowning in complex information overload. (Neonatologist) It gives families something they can take away and read that aligns to what they’ve been told in that (bereavement counselling) setting. (Neonatologist) I think it would be extremely useful because when you mention to families they sort of close down and they forget the questions they might want to ask but having a resource that they can go to when they maybe deal, come to maybe deal with a little bit more terms with the information they’ve just been given, a resource that you can give them and say, when you feel up to it, have a read through this. I actually think that would be extremely beneficial. (PICU nurse) Sometimes there’s not a huge amount of time between then and when an intubation happens for them to read … so maybe when they can read it at a point when they’re a little bit kind of calmer then at least there’s some sort of information there and if they come to it, they can go, “Oh, I’ve read that book and I should maybe pick that up again”. (Organ and tissue donation nurse coordinator)
	**Sub-theme 3: ‘I’m not alone in this’** I like that there’s some stories and I really like the quotes and I think that’s really helpful for people to say, yeah that’s what I’ve been thinking or that’s been my experience or just hearing something, being reminded that I’m not alone in this. Other people have walked this journey. (Chaplain) It would help those who are struggling to make a decision – seeing others have gone through it would help. (PICU nurse) I think it just made it a bit more personalised it sort of opened it up that, you know what, other families go through this as well and this is how they dealt with it. (PICU nurse) I think it’s really good. I think the inclusion of the post-it notes with the families’ feedback is really good because it makes it feel much more like it isn’t just the doctors telling you what they think. (Educational play therapist)	

### Theme 2: Not just for families

The second main theme, *Not just for families*, encapsulated the finding that the *Caring Decisions* resource was viewed as a valuable tool for health professionals themselves, because it informed them about what to raise with families (See Table 2 for example quotes).

**Table 2:** Theme 2: Not just for families

**Sub-theme 1: A guide for staff** I have conversations with our nursing staff about what to say to families and stuff like that whereas this resource would be good. I actually think it’s not just about, this resource isn’t just good for families, actually, it’s really good for nursing staff as well or maybe even all health professionals (PICU nurse) From a nursing perspective, I think it’s really good because it helps you, like when you read it, it’s like well this is how clear you can be about the information. … It gives you sort of like a guide, like you know this is what the family’s been given, so you can use that as a guide and knowing what other families found useful as well. (PICU nurse) We don’t actually have anything we can get our hands on and say, here’s something to read and think about it. (Organ and tissue donation nurse coordinator) It’s an experience you learn on the ground and some people haven’t learnt it well. Not necessarily those in the intensive care. In intensive care, by exposure, you’d have to have come across it but generally a lot of doctors don’t do it that well. (Cardiologist) I’ve not, it’s not something that we’re ever taught as such. I mean the times I’ve learnt it has been as a registrar and as a fellow watching my mentors do it. (Cardiologist) Good resource for nurses. (Notes from interview with PICU nurse) I think it might [be good for] for people that are new to the ICU setting so haven’t, you know, are interested in bereavements and death and dying and that it gives them information that they might not want to ask to colleagues. (PICU nurse) The thing I like about it though is it does focus attention on the issue and it gives clinicians and bedside staff a shared language, as you say, because we all do have slightly different ways of articulating things and, whilst we all strive for clarity, I’m not sure that that’s always achieved and I think if the resource was to become part of routine clinical practice, it would do a lot to standardise the way that we approach this. (Neonatologist)
**Sub-theme 2: Processes of Communicating - When to give the resource?** I think what I’ll do each time is I’ll re-read the book… And then talk to the family and perhaps giving them the whole book but it would be very dependent on the individual and the circumstance. (Paediatrician) It’s very difficult because you don’t want to introduce it too early but if someone’s in that distressed a state, I don’t know how much they register with because this is very logical you know. (Educational play therapist) So I think in cases like that where it’s less than 24 hours from admission to declaration of death I think that’s probably a bit too soon, it’s a bit too much trauma happening for them to read it. I think where they’re going to be here for a few days and you’ve got the time to sort of introduce something they can think about it and feel better. (PICU nurse) Yep, I think you could give it to them as part of the conversation (about EoL) or maybe if that conversation was happening about, you know, this is what we think might happen, so giving it to them so that when you got to that point where you were actually having a conversation about life limiting or conditions and stuff, they already had that resource. (PICU nurse) I think it’d be great in those cases where children who are slowly deteriorating and it looks like we’re heading towards the point where potentially an end of life discussion is going to be held or some parents come in and, for example, like a head injury. We might call them for 3 days, we warn them and scan and the parents kind of know from the beginning that the outcome’s probably not going to be very good but we’re not going to decide for a few days and I think with them, something like this again would be great. (PICU nurse) In ICU, I wouldn’t start a conversation about death and dying and saying, and here’s a pamphlet. So I think that it’s a bit beyond that stage. (Paediatric intensivist) I think the best time for people to read this book is not at the time they walk into ICU or at one of our family meetings. Ideally it’s a year beforehand. (Cardiologist) My first thought was this would be a useful resource if it was clear that a child had a condition which would lead to death eventually, where you might want the family to start to or you might have a discussion with the family about the possibility that the child’s going to die and they could read it and then come back to it closer to the time when or if that happened. (Paediatrician)
**Sub-theme 3: Processes of Communicating - Who should be involved?** I thought I read somewhere that it was just the doctor that would give it. … I would think it would be appropriate for the care manager or someone from the healthcare team. (Chaplain) I think overall you get the sense that and I don’t know the context in which you’re giving it to people but it’s very much about helping families to feel OK with stopping treatment. That’s the sort of vibe I got with it that this is what you’d say if you and if the health professionals were feeling like that with the time this is when you’d give to them, not when it was still a question mark about whether or not, do you know what I mean? (Educational play therapist) I’d definitely feel there’d be situations where the nurse would be appropriate to give it or to talk to the doctors about the doctors giving it. You know, some families respond best to the bedside nurse because the bedside nurse is there for 8 to 12 hours at the time whereas some of them only really trust their medical team and if that’s the case, then the medical team would be the most appropriate because some of them find the social worker the most consistent to being throughout their admission so the social worker would be the most appropriate. (PICU nurse) Probably the social worker is the best way to get it in here, and I think some consultants would probably be much more open and receptive to it than others. (PICU nurse) I think possibly one of the best people in a lot of our situations would probably be our social workers because the social workers really often end up really talking in laymen’s terms with the nurses and the families so they’ll find out from us where the families are at and how the patient’s going but then they sit with them on a different level. It’s often, I don’t know how to describe it, it seems like it’s a less medically driven discussion and I think a lot of this terminology is trying to break that barrier between medical and laymen’s terms whereas but I think the social workers would be able to clue in when that would be most appropriate. (PICU nurse) I wonder who the best person to actually give them the booklet is. It won’t always be one of the consultant doctors. I suspect the role of our, particularly in the ICU, the care co-ordinators. I suspect they would, for many families, be the best people to be handing out this booklet I reckon. *(Interviewer: And is that because you think that the families sort of see the role of the doctors slightly differently or the intensivist and the cardiologist?)* Maybe. I think the care co-ordinator spends more time with the family. They definitely spend more time with the family so they’d be about, often they would find an appropriate time to sort of hand out the booklet and I know they often try and gauge where the family’s at prior to meetings to help us guide the content of meeting and to have a feel for the inside story. (Cardiologist) So having something is better than nothing and we’re relying at the moment on the personality of the person having the conversation. (Paediatric intensivist)
**Sub-theme 4: Processes of Communicating - How to give the resource?** They [patients] would have to make the approach to the subject with me and it’d have to be at that phase of having those conversations to be able to give it to them but I would feel comfortable giving it. (PICU nurse) Within most wards, is an area where parents can access written information for various things, and if it were to be sat on a rack with various things and if it were to be a rack in a family or parent room, it’s very clearly titled “Caring decisions – a handbook for parents facing end of life decisions for their child”, if anyone didn’t want the shock, then they wouldn’t pick it up. But for people that were interested, even though they might not be in that situation immediately, it would still allow them the benefit of having the information ahead of perhaps having the meeting where the information. … I also think perhaps the availability of the resource could be something the parents could self-regulate. (Neonatologist) Look, I agree that it’s hard to introduce until you have that discussion and I think if there’s a way to have it available to parents so they know it’s there if they need it. I don’t know how you’d, I don’t know if there’s maybe like an orientation to the hospital leaflet that parents are given that you could say on it, you know, if you think if you’re getting towards a point, there’s this resource available to you. (PICU nurse) Yes. I can see myself, I would give it to the families I thought it would help the most and give it to them and say, you don’t even have to open this book if you don’t want to but put it in your bag and keep it, you just never know. You might want to open it and I would also, so many of our families are computer savvy, I’d say, if you don’t want to open the book, at least go to the website because there’s lots of things there and then I’ve given them the opportunity and it’s up to them. (PICU nurse)

The three further subthemes focused on processes of communication including timing, people involved and how to begin the conversation. The first sub-theme of theme 2, *A guide for staff*, arose from considerable participant discussion about a lack of resources for them when dealing with end-of-life decisions, as well as a lack of formal training for healthcare staff who talk to parents about such decisions. Participants expressed the view that having such a resource would help them communicate information more effectively. In the same way that it gives a language to families, it also provides a model for staff about how to communicate about end-of-life decisions and issues, both inter-professionally and with patients’ families.

The last three sub-themes of theme 2 illustrate how the handbook acted as a trigger for clinicians to think more broadly about *processes of communication* in this area of their practice. Being asked to consider the contents of both the handbook and online resource led interviewees to discuss when, how, and why they would use such a resource, who would disseminate it and how it related to their current processes of communication with families and with each other.

Sub-theme two, *When to give the resource?*, was a key topic discussed by participants. Although participants believed the handbook could provide accessible information, including giving parents a way to think about treatment decisions for their seriously ill child, they also spoke of feeling uncertain about the most appropriate time to pass on this information. Some participants spoke of the need to wait until parents seemed to be ready to consider this information because if parents were given this information when they were highly anxious or in shock, it might add to the burdens they were already experiencing.

Several participants expressed the opinion that the resource was probably not appropriate for distribution in acute situations, in ICU for instance, where a child’s death was imminent and/or unexpected, and parents had very little time to make a decision and process rapidly developing devastating events. However, they thought that where a child’s health was slowly deteriorating and where there was a possibility of planning ahead, it would confer benefits.

Considerations about the most appropriate time to provide parents with the resource as well as the best approach to communicating information to parents was also linked to diagnostic and prognostic uncertainty. Interviewees spoke of wanting to assist parents to move from a denial of the facts to a greater understanding of the complexity of the prognosis and felt that the resource may assist in this process.

There was agreement that the clinical judgement of the treating team or the individual clinician dictated what information to communicate and when to communicate it, with less agreement about who was the most appropriate person to talk with parents (See quotes in Sub-theme 3: *Who should give the resource?*).

The third communication process subtheme concerned ‘how’ the resource could be disseminated (Sub-theme 4: *How to give the resource?*). Some participants thought leaving the book in the ward, including it in an admission pack or referencing it in a pamphlet as part of standard and general information might ‘normalise’ this type of information and enable parents to take in and digest the information before they were personally facing such decisions.

### Barriers to implementation

In general, the feedback from participants relating to the layout and presentation of the resource was positive:

When I read it I was like, this is really great! It’s compact, small, clear. It’s not, like even the colours and stuff, like I know that’s something really little but they’re not intimidating. … I thought it was calming. (Nurse)

However, some participants raised possible barriers to the use of the booklet, arising from the language used. For example, some felt that the reading level was too advanced. In addition, the size of the font in the handbook was felt to be too small by one participant. There were also some preferences regarding the language used:

The only thing I didn’t like is when people use the term “vegetative”. I just, personally, I hate that term. I think it’s old school. (Int14)

Other terms that drew criticism for being misleading or not accurate enough, were ‘comfort treatment’ and ‘stopping treatment’ . Some participants also mentioned that it would be preferable to have translations of the resource available for families with limited English proficiency.

## Discussion and Conclusion

### Discussion

This research aimed to evaluate the acceptability of the parents’ resource *Caring Decisions* and to ascertain whether healthcare professionals would be willing to incorporate the resource into standard practice. In accordance with the findings of the pilot evaluation study [34], all of the health professionals interviewed viewed the resource positively and indicated that they saw a need for it and would use it themselves, or refer people to it.

Interviewees also reiterated and elaborated on other key findings from Xafis et al. [26], such as a general lack of availability of resources of this type, the usefulness of providing parents with a language that could facilitate further communication, and a perception that ascertaining the right time to distribute or make parents aware of the resource is delicate and, if not done well, could present some difficulties. Parents have also expressed the view that such information should only be provided when parents are ready to receive it [50].

The fact that parents are able to take the handbook away and read it in their own time because they may be too shocked and traumatised to take in all the information they are presented with during meetings has been previously recognised [51, 52]. Written materials have also been shown to be helpful in improving parents’ understanding of basic medical information about their child [53].

The research findings also resonate more broadly with ideas about workplace learning and cultural shifts which are necessary to change patterns of communication. All participants agreed there was a need for such resources but they differed in their opinions of when or how the resource should be given to parents, and also who should talk to the parents. These challenges are reflective, not only of difficulties in communication due to the subject matter [54], but also of the negotiation of hierarchies and associated communication structures and routines within a hospital or care setting [55], where decisions involve high stakes, and outcomes often may be uncertain. Integrating new resources, such as the *Caring Decisions* handbook and web-based information into complex health environments requires on the one hand, individual agency and intention to use the booklet and speak to parents, and on the other, processes of negotiation with other team members to develop shared meanings about and authority to use the resource [56, 57]. Research suggests parents would like as much information as possible, [8, 13–15] and according to this current research and previous studies about this resource [34], clinicians are supportive and find the information useful. However this research also highlights some challenges that may still arise when integrating these ideas and practical suggestions into the workplace.

Writing from a broader perspective, Frenk [58] suggests ‘unprecedented teamwork’ is required for health professionals to coordinate care across time and space. Closer to the paediatric intensive care environment, John Lantos [59] describes the type of teamwork necessary in having end-of-life conversations in a more nuanced way, suggesting there is a need to take ‘some course of action that violates neither the values of the dying patient nor the values of the survivors, who must live with the memory of the action’ (p.97). According to this research, the *Caring Decisions* resources are likely to assist in clarifying values and providing a shared language between parents and clinicians. However to achieve this, by helping parents to clarify their own values and to talk with clinicians about their child, the resource must become part of the practical wisdom and lived experience [60] of the paediatric critical care environment. This is the next challenge.

### Conclusion

This research indicates a willingness to adopt the *Caring Decisions* resources in clinical practice situations, where decisions to withdraw or withhold treatments need to be made by parents and healthcare professionals caring for these children. The research demonstrates that the resources were considered to be useful in improving discussions with parents and to fill a gap in parents’ resources that discuss both clinical aspects but also, importantly, ethical issues that are relevant to such end-of-life decisions. Opinions on the ‘who, when, and how’ of dissemination of the resource, however, were divergent among the health care staff interviewed, indicating that implementation of the resource in the hospital context may not be straightforward and raises questions that may require explicit discussion and negotiation within the health care team.

### Limitations of the study

This research focused exclusively on healthcare professionals’ views from one paediatric public hospital in Australia. This limits the transferability of the findings to other contexts, however the links between emergent themes in this research and studies of end-of-life communication in paediatric settings assists the reader to evaluate the relevance and fit to their context [61]. In addition, no parent interviews were conducted. Interviewing parents would be very helpful and informative and would add to the information already collected both through the consultation process and the pilot evaluation study [34]. This is a further project to be undertaken.

### Practice implications

This study confirms the value of and need for the *Caring Decisions* resources in the eyes of the clinician. To ensure adequate uptake and use of the handbook and website, discussions with the health care team around *when* to use it, and also *how* to use it, and *who* should give it to families, will be essential. In addition, a further beneficial initiative would be to create translations of the resource for non-English speaking families, incorporating the appropriate cultural considerations for each group.

## Appendix 1: Interview Guide

Introductory comments

- Thank you for making the time to talk with me today about the ‘caring decisions’ booklet and web-based resource
- Have you read the information about the interview in the PIS?
- Have you signed the consent form?

The goals of this interview are to ask whether you think this booklet and the accompanying online resource would be helpful in your conversations with parents and whether you would be willing to provide a copy to parents?

1. First, can I very briefly ask you about your role in caring for critically ill children?
  - What is your position here at RCH?
  - How long have you been working in this area?
  - Do you have a particular approach (which you have found to be helpful) when discussing treatment withdrawal or decisions about life sustaining treatments with parents?
2. Have you had a chance to read this handbook and the more comprehensive web resource?
3. What are your thoughts about the parent handbook and web resource?
  - What sorts of resources do you draw from when discussing end of life care and decisions with parents?
4. Would the resources be helpful in your conversations with parents?
5. Do you think parents would use these resources?
  - Would they be helpful for them?
  - Do you think they would prefer the web-based version which has more detailed information?
6. Are there particular parents or situations that you can think of where this booklet/web resource would /would not be suitable?
7. In what circumstances would you be willing/not willing to provide a copy to parents?
8. How widely do you think this resource should be disseminated (if at all)?
9. What improvements could be made?
